# The Effect of Maternal Pertussis Immunization on Infant Vaccine Responses to a Booster Pertussis-Containing Vaccine in Vietnam

**DOI:** 10.1093/cid/ciw551

**Published:** 2016-11-02

**Authors:** Kirsten Maertens, Thi Thu Ha Hoang, Trung Dac Nguyen, Raïssa Nadège Caboré, Thi Hong Duong, Kris Huygen, Niel Hens, Pierre Van Damme, Duc Anh Dang, Elke Leuridan

**Affiliations:** 1 Centre for the Evaluation of Vaccination, Vaccine and Infectious Diseases Institute, University of Antwerp, Belgium; 2 Bacteriology Department, National Institute of Hygiene and Epidemiology, Hanoi, Vietnam; 3 National Reference Centre Bordetella, National Reference Centre Toxigenic Corynebacteria, Service Immunology, Scientific Institute of Public Health, Brussels; 4 Interuniversity Institute for Biostatistics and Statistical Bioinformatics, Hasselt University; 5 Centre for Health Economics Research and Modeling Infectious Diseases, Vaccine and Infectious Disease Institute, University of Antwerp, Belgium

**Keywords:** pertussis, vaccination in pregnancy, maternal antibodies

## Abstract

***Background.*** Maternal vaccination with an acellular pertussis (aP)–containing vaccine is a recommended strategy in a growing number of industrialized countries, to protect young infants from disease. Little is known on the effect of this strategy in low- and middle-income countries. Following a previous report on the effect of adding a pertussis and diphtheria component to the tetanus vaccination program in pregnant women in Vietnam, we report on infant immune responses to a booster aP vaccine dose in this randomized controlled clinical trial.

***Methods.*** Thirty infants of Tdap (tetanus, diphtheria, and acellular pertussis)–vaccinated pregnant women and 37 infants of women vaccinated with a tetanus-only vaccine received a fourth aP-containing vaccine dose in the second year of life. Blood was taken 1 month after the fourth infant dose. Immunoglobulin G (IgG) antibodies against pertussis toxin (PT), filamentous hemagglutinin (FHA), pertactin (Prn), tetanus toxoid (TT), and diphtheria toxoid (DT) were measured using commercially available enzyme-linked immunosorbent assays (ELISA).

***Results.*** One month after the booster dose, significantly lower antibody titers were measured in the Tdap group for anti-TT IgG (*P* < .001) only. Anti-DT IgG, anti-PT IgG, anti-Prn IgG, and anti-FHA IgG antibody titers were comparable for both groups. A rise in antibody concentrations was elicited for all (except DT) antigens after boosting.

***Conclusions.*** The present results indicate that the blunting of infant pertussis responses induced by maternal immunization, measured after a primary series of aP vaccines, was resolved with the booster aP vaccine dose. These results add to the evidence for national and international decision makers on maternal immunization as a vaccination strategy for protection of young infants against infectious diseases.

In 2014, global coverage of the 3 primary infant DTP (diphtheria, tetanus, pertussis) vaccine doses was as high as 86%. Despite these successful global pertussis vaccination programs, the disease remains an important public health issue, causing an estimated 63 000 deaths in children <5 years of age (2013) [1]. Mainly young infants, too young to be protected by the currently available vaccines and vaccination schedules, are prone to severe pertussis disease and have the highest hospitalization and complication rates among the population [2].

Vaccination during pregnancy has been implemented in national vaccination programs to elicit high titers of maternal antibodies, as a means to protect young infants from disease [3–5]. High titers of maternal antibodies induced by maternal vaccination have already been shown to interfere with the infant humoral immune response on primary acellular pertussis (aP) vaccination [3–6]. This blunting effect ceased after a fourth aP vaccine dose at the age of 12 months in a randomized controlled trial conducted in the United States [3]. Yet, few data are available on infant immune responses to a fourth pertussis vaccine dose using different intervals in infant immunization schedules.

In Vietnam, infant pertussis vaccination with whole-cell pertussis (wP) vaccines started in 1985. Prior to that, the incidence of pertussis was up to 84.4 per 100 000 (1984) [7]. Overall, the reported incidence is now relatively low. In 2015, based on clinical criteria, 309 pertussis cases were reported, resulting in an incidence of 0.3 per 100 000 (personal communication, National Institute of Hygiene and Epidemiology [NIHE], Vietnam). In the period 2011–2013, >50% of the cases occurred in infants <1 year of age. In 2014, 92 of 102 pertussis cases were reported in infants aged <6 months [8].

The World Health Organization (WHO) recommends the use of wP vaccines within the Expanded Programme on Immunization (EPI) [9] whenever a 3 + 1 infant-only schedule is used. National programs currently administering wP vaccination should continue to use wP vaccines for the primary vaccination schedule. A switch from wP to aP vaccines for primary infant immunization should only be considered when additional boosters or maternal immunization are included in the national immunization schedule [1].

We have previously reported on the effect of high titers of maternal antibodies on the primary infant immune responses to aP infant vaccines in Vietnam, after maternal vaccination during pregnancy with a combined tetanus, diphtheria, and aP (Tdap) vaccine (Adacel, Sanofi Pasteur, Canada) [4]. The present article assesses the possible remaining blunting effect of maternal immunization with the infant humoral immune responses after a fourth aP-containing vaccine dose, administered in the second year of life.

## MATERIALS AND METHODS

A randomized controlled study was conducted in accordance with the Helsinki Declaration, Good Clinical Practice, and the procedures established by Vietnamese law. Ethical approval was obtained (NIHE, Vietnam: No. 05IRB-120412; No. IRB-VN1059-02; and Ministry of Health: No. 978/CN-BYT-131112). Written informed consent was signed by all participants and both parents of the infants. Extended information on material and methods has been reported previously [4].

Participating children were included in either a Tdap group—that is, children born from women vaccinated with an aP-containing vaccine (Adacel) between 18 and 36 weeks of pregnancy—or a tetanus toxoid (TT) group—that is, children born from women vaccinated with a tetanus-only vaccine (TT-Institute of Vaccine and Biological Products [IVAC], Hanoi, Vietnam) during pregnancy, as recommended within the EPI.

Within the present study, all infants received Infanrix Hexa (GSK Biologicals, Rixensart, Belgium) for primary vaccination at the age of 2, 3, and 4 months [4]. A fourth Infanrix Hexa dose was planned to be administered at the age of 18 months. Due to delay in the approval of the ethics committee, resulting in a suspension of approximately 4 months, some of the infants within this study were already vaccinated with a wP-containing vaccine (DTP) within the EPI, whereas most children received Infanrix Hexa as a booster dose in the second year of life.

From all children in the study, data on health status and growth parameters were collected at the moment of the fourth vaccine dose.

### Study Vaccines

Infants received either the hexavalent vaccine Infanrix Hexa (GSK Biologicals, Belgium) or a DTwP (diphtheria, tetanus, wP) vaccine (IVAC). Infanrix Hexa contains 10 limit of flocculation units (Lf) TT, 25 Lf diphtheria toxoid (DT), 25 µg pertussis toxin (PT), 25 µg filamentous hemagglutinin (FHA), and 8 µg pertactin (Prn) plus inactivated poliovirus, hepatitis B surface antigens, and *Haemophilus influenzae* type B polysaccharide. The DTwP vaccine used in the study contains purified diphtheria anatoxin (30 International Units [IU]), purified tetanus anatoxin (60 IU), and inactivated wP (4 IU) adsorbed by aluminum phosphate.

### Study Procedures

All infant vaccines were administered at the Commune Health Center (CHC) during the second year of life [4]. Blood samples were collected from the infants 1 month after the fourth vaccine dose. All blood samples were collected at the CHC and transported to the Ha Nam Preventive Medicine Center on the same day. Samples were centrifuged and stored at −80°C. All samples were monthly sent to the Department of Bacteriology at NIHE.

### Laboratory

All frozen samples were transported to the Scientific Institute of Public Health in Brussels, Belgium, and tested with commercially available enzyme-linked immunosorbent assay (ELISA) kits. The Virion/Serion kit (ANL, Copenhagen) was used to detect anti-PT immunoglobulin G (IgG) antibodies and the Euroimmune ELISA kit was used to detect anti-FHA and anti-Prn IgG antibodies. Anti-TT and anti-DT IgG antibodies were detected using the Virotech/Sekisui ELISA kit. Serum samples were tested at a dilution of 1:100. ELISA results were expressed in international units per milliliter (IU/mL), using respective WHO standards (National Institute for Biological Standards and Control [NIBSC] code 06/140 for pertussis, NIBSC code TE-3 for tetanus, and NIBSC code 00/496 for diphtheria). For pertussis, these international units are equivalent to the ELISA units of the Center for Biologics Evaluation and Research, US Food and Drug Administration [10]. The lower limit of detection of the assays was 0.7 IU/mL for PT, 1 IU/mL for FHA, 3 IU/mL for Prn, 0.01 IU/mL for TT, and 0.03 IU/mL for DT.

To guarantee the reliability of the results, an international independent validation was performed at the Canadian Center for Vaccinology in Halifax, Canada [4, 5, 11].

For pertussis, a protective threshold of antibodies (correlate of protection) is not known [12]. However, low antibody concentrations are correlated with susceptibility to pertussis infection [13, 14]. For tetanus and diphtheria, the correlate of protection is defined as 0.1 IU/mL for tetanus and 0.01–0.1 IU/mL for diphtheria.

In this paper, blunting of the immune response after the fourth vaccine dose among infants was defined by the authors, similarly to a previous publication [4], as a significantly lower geometric mean concentration (GMC) of specific IgG antibodies, measured 1 month after the fourth vaccine dose in the Tdap group compared to the TT (control) group.

### Statistical Analysis

The initial sample size calculation was based on previous results [15]; a population of 50 subjects in each study arm would be sufficient to detect significant differences in antibody titers of IgG in cord and newborns. No additional sample size calculation has been performed, due to a lack of data for the postbooster time point at the conception of the study. The original aim was to vaccinate all infants with an aP-containing vaccine for their fourth vaccine dose. Due to unforeseen circumstances, some children were vaccinated with a wP-containing vaccine, resulting in a smaller number of aP-vaccinated infants in both study groups, mainly in the Tdap group. Therefore, the study might be underpowered because of these unforeseen circumstances.

Disease-specific antibody GMCs and 95% confidence intervals (CIs) were calculated at each time point in both study groups. Descriptive analyses were performed to identify possible differences between both study groups. Statistical tests included parametric tests: (paired) *t* tests and *χ*^2^ tests and their nonparametric alternatives: (paired) Wilcoxon tests and Fisher exact tests whenever the underlying assumptions of the parametric tests were violated (ie, normality and sparseness assumptions, respectively) [16, 17]. Linear regression models were used to identify characteristics that could potentially impact infant antibody titers 1 month after the administration of a fourth vaccine dose.

The analysis was performed using SPSS statistical software version 23.0. A 2-sided *P* value <.05 was considered statistical significant.

## RESULTS

### General Characteristics of the Study Population

Characteristics of the mother–infant pairs until 5 months after delivery as well as exclusion criteria at baseline have been described in a previous publication [4]. Children were born between 22 February 2013 and 7 October 2013. After birth, 51 children were included in the Tdap group and 48 children in the TT group. After the primary series of 3 aP-containing vaccines, 15 children from the Tdap group and 4 children from the TT group were vaccinated “not according to protocol” with a wP vaccine as a fourth vaccine dose. Due to loss to follow-up, 6 additional children from the Tdap group and 7 additional children from the TT group were excluded from the study. In the end, 30 infants were included in the Tdap group and 37 infants in the TT group for analysis of the postbooster responses.

Infants were vaccinated with a fourth aP-containing vaccine (Infanrix Hexa) between 4 April 2015 and 10 May 2015 at a mean age of 22.18 months (range, 18.5–24.7 months). All children were in good health at the moment of vaccination. Blood samples were taken on average 30.2 days (range, 30–33 days) after the fourth vaccine dose between 7 May 2015 and 10 June 2015.

Comparing demographics between children from the Tdap group and children from the TT group, a significantly smaller interval between vaccine dose 3 and vaccine dose 4 was found in the TT group (*P* = .010; Table 1).

**Table 1.** Demographic Characteristics of Infants at Booster Vaccination

Characteristic	Tdap Group	TT Group	*P* Value
No. (included infants)	30	37	
Infant sex, No. (%)			
Male	20 (66.7)	21 (56.8)	.458
Female	10 (33.3)	16 (43.2)	
Mean weight, kg (SEM)	10.75 (0.20)	10.59 (0.18)	.562
Mean length, cm (SEM)	83.20 (0.59)	81.71 (0.58)	.078
Mean age at vaccine dose 4, mo (SEM)	22.18 (0.27)	21.44 (0.29)	.071
Mean age at blood sample 1 mo after fourth vaccine dose, mo (SEM)	23.18 (0.27)	22.43 (0.29)	.069
Mean interval between vaccine dose 4 and blood sample 1 mo after fourth vaccine dose, mo (SEM)	0.99 (0.01)	0.98 (0.00)	.175
Mean interval between vaccine dose 3 and vaccine dose 4, mo (SEM)	17.44 (0.17)	16.58 (0.26)	.010

Abbreviations: SEM, standard error of the mean; Tdap, tetanus, diphtheria, and acellular pertussis vaccine; TT, tetanus toxoid.

The clinical history of the participants did not identify any pertussis disease case in the infants nor in the households during the entire study period.

### Laboratory Results

Table 2 provides an overview of the GMCs of IgG antibodies to TT, DT, and 3 pertussis-specific antigens in the sera of all infants at delivery, before the start of the primary pertussis vaccination, 1 month after the primary pertussis vaccination and 1 month after the administration of the fourth aP-containing vaccine dose during the second year of life.

**Table 2.** Antibodies to Tetanus Toxoid, Diphtheria Toxoid, Pertussis Toxin, Filamentous Hemagglutinin, and Pertactin at Delivery, Before Primary Vaccination, 1 Month After Primary Vaccination and 1 Month After the Fourth Acellular Pertussis–Containing Vaccine Dose in Both Groups of Infants

Antigen Included in the Infant Vaccine	Cord		Before Primary Vaccination		1 mo After Primary Vaccination		1 mo After Fourth Vaccine Dose	
	Tdap Group	TT Group	Tdap Group	TT Group	Tdap Group	TT Group	Tdap Group	TT Group
No. of samples	49 (50 for anti-PT)	47 (46 for anti-FHA)	45 (51 for anti-TT and anti-DT)	48 (35 for anti-Prn and anti-FHA and anti-PT)	35 (51 for anti-TT)	35 (49 for anti-TT)	30	37
Tetanus toxoid, IU/mL	2.2 (1.5–3.2)	1.1 (.6–1.9)	0.36 (.2–.6)	0.25 (.2–.4)	1.5 (1.3–1.8)	1.0 (.8–1.2)	2.7 (2.4–3.1)	4.2 (3.7–4.7)
*P* value	.046		.329		.001		<.001	
Diphtheria toxoid, IU/mL	0.24 (.1–.4)	0.05 (.04–.07)	0.14 (.1–.2)	0.05 (.04–.06)	1.96 (1.62–2.3)	2.80 (2.48–3.12)	2.0 (1.6–2.4)	2.3 (2.1–2.6)
*P* value	<.001		<.001		<.001		.187	
Pertussis toxin, IU/mL	21 (16–28)	7.2 (5.6–9.4)	4.2 (2.9–5.9)	0.8 (.5–1.3)	70 (58–84)	67 (53–84)	129.0 (97.5–170.7)	133.7 (106.6–167.6)
*P* value	<.001		<.001		.753		.845	
Filamentous hemagglutinin, IU/mL	93 (65–133)	27.6 (20.9–36.7)	59 (48–73)	23.1 (19.7–27)	77 (66–90)	66.6 (56–78)	161.3 (134.1–193.9)	181.7 (160.3–206.0)
*P* value	<.001		<.001		.198		.285	
Pertactin, IU/mL	124 (86–179)	13.9 (10.5–18.2)	46 (32–66)	7.8 (6.6–9.4)	83 (65–104)	132.6 (104–168)	159.0 (141.2–179.0)	187.1 (163.8–213.6)
*P* value	<.001		<.001		.006		.085	

Data are presented as geometric mean concentration (95% confidence interval) unless otherwise indicated.

Abbreviations: DT, diphtheria toxoid; FHA, filamentous hemagglutinin; Prn, pertactin; PT, pertussis toxin; Tdap, tetanus, diphtheria, and acellular pertussis vaccine; TT, tetanus toxoid.

One month after a primary series of 3 doses of the hexavalent aP vaccine, significantly lower antibody titers were observed in infants from the Tdap group compared with infants from the TT group for anti-Prn IgG (GMC, 83 [95% CI, 65–104] vs 132 [95% CI, 104–168]; *P* = .006) and anti-DT IgG (GMC, 1.96 [95% CI, 1.62–2.30] vs 2.80 [95% CI, 2.48–3.12]; *P* < .001) antibodies. For anti-TT IgG, anti-PT IgG, and anti-FHA IgG, however, comparable but higher antibody titers were reported in infants from the Tdap group compared with infants from the TT group [4].

One month after the administration of the fourth aP-containing vaccine, GMCs to anti-TT IgG (GMC, 2.7 [95% CI, 2.4–3.1] vs 4.2 [95% CI, 3.7–4.7]; *P* < .001) were significantly lower in infants from the Tdap group compared with infants from the TT group. For anti-DT IgG, anti-PT IgG, anti-FHA IgG, and anti-Prn IgG, comparable but lower antibody titers were found in infants from the Tdap group compared to infants from the TT group.

**Figure 1.**
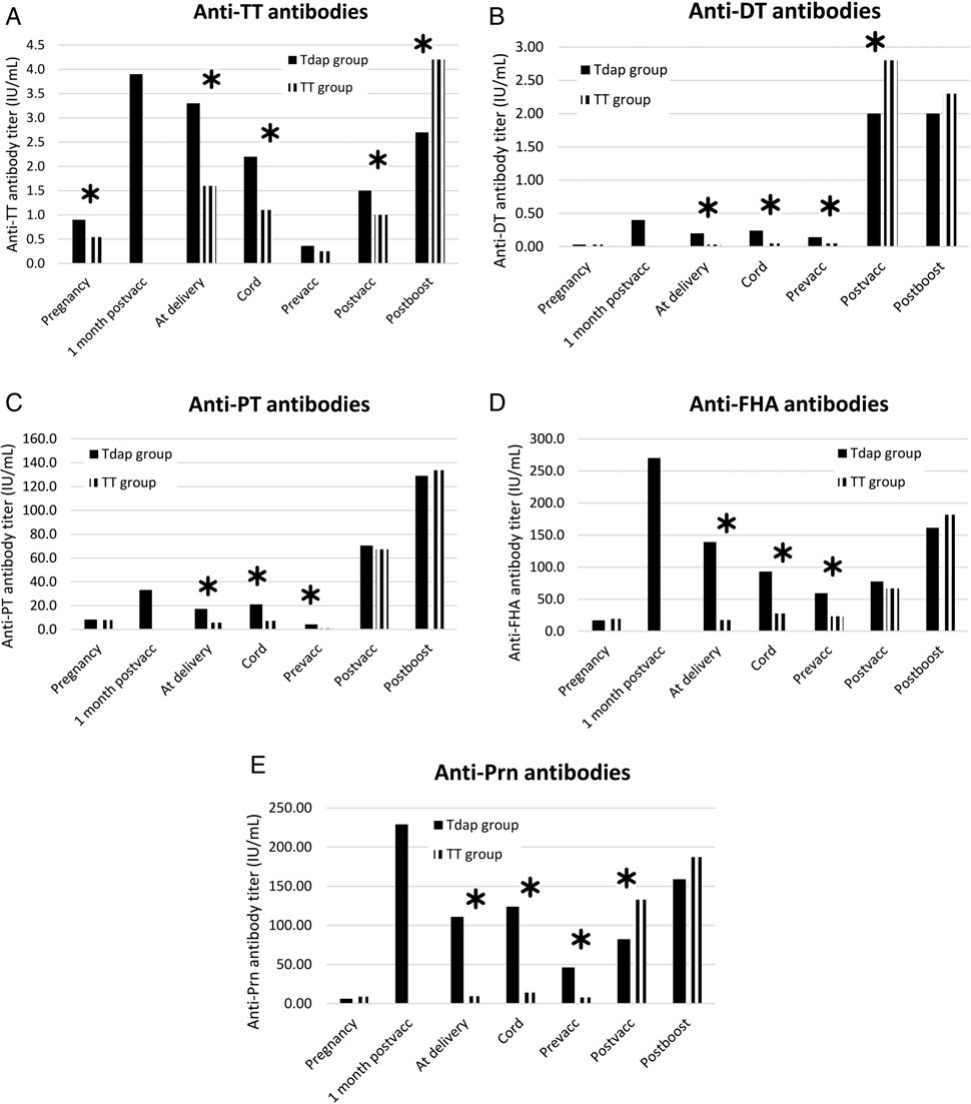
Geometric mean concentration for antibodies to tetanus toxoid (TT), diphtheria toxoid (DT), pertussis toxin (PT), filamentous hemagglutinin (FHA), and pertactin (Prn) in both groups of women and infants at all time points. *A*, Anti-TT antibodies. *B*, Anti-DT antibodies. *C*, Anti-PT antibodies. *D*, Anti-FHA antibodies. *E*, Anti-Prn antibodies. *Significant differences between the Tdap and the TT group.

Figure 1 shows the GMCs for antibodies to TT, DT, PT, FHA, and Prn at all time points in both study groups, including the data that have been published before [4]. For all antigens, except for anti-TT IgG, a significant correlation was found between the antibody titers 1 month after the primary vaccination and 1 month after the fourth vaccine dose.

### Results From the Regression Analysis

We only report the significant influences of variables on the antibody titers 1 month after the booster infant dose. The variables used in the linear regression analysis are described in Table 1.

In the TT group, a significant influence of the interval between vaccine dose 3 and 4 (16.58 months [standard error of the mean {SEM}, 0.26 months]) on the anti-FHA IgG (*P* = .028) was found; the antibody titer decreases with an increasing interval between vaccine dose 3 and 4. In addition, a significant influence of sex (*P* = .013) on the anti-DT IgG was found. Male infants have a significantly higher antibody titer compared to female infants.

In the Tdap group, no significant influences of any variable on the antibody titer was found.

### Additional Results on wP-Boosted Infants

As only 4 children in the TT group were boosted with a wP-containing vaccine, the sample size of this group was insufficient to analyze the results separately.

Within the Tdap group, 15 children received a wP-containing vaccine as a fourth vaccine dose. A significantly higher mean age at vaccine dose 4 (22.18 months [SEM, 0.27 months] vs 20.81 months [SEM, 0.43 months]; *P* = .007) and a significantly higher mean interval between vaccine dose 3 and 4 (17.44 months [SEM, 0.17 months] vs 15.24 months [SEM, 0.29 months]; *P* < .001) were calculated in aP-vaccinated infants compared with wP-vaccinated infants, due to the delay in institutional review board approval for the use of aP-containing vaccines for booster. As a consequence, a significantly lower interval between vaccine dose 4 and blood sampling (0.99 months [SEM, 0.01 months]) vs 3.16 months [SEM, 0.20 months]; *P* < .001) was calculated in aP-vaccinated infants compared with wP-vaccinated infants within the Tdap group (Supplementary Table 1).

**Figure 2.**
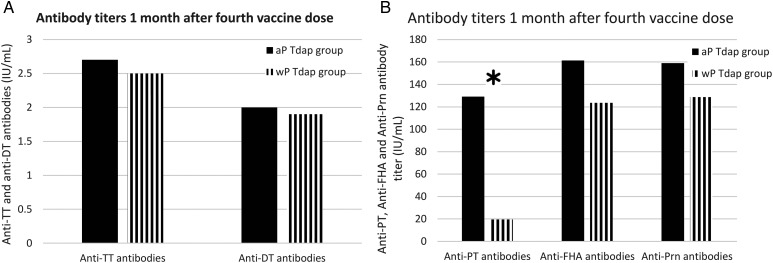
Geometric mean concentration for antibodies to tetanus toxoid (TT), diphtheria toxoid (DT), pertussis toxin (PT), filamentous hemagglutinin (FHA), and pertactin (Prn) in infants from the tetanus, diphtheria, and acellular pertussis vaccine (Tdap) group after the administration of the fourth acellular pertussis (aP)– or whole-cell pertussis (wP)–containing vaccine dose. *A*, Anti-TT and Anti-DT antibodies. *B*, Anti-PT, anti-FHA, and anti-Prn antibodies. *Significant differences.

Overall, higher antibody titers were found in fully (4 doses) aP-vaccinated infants, yet only for anti-PT IgG, these antibody titers were significantly higher in the aP-boosted infants (GMC, 129 [95% CI, 97.5–170.7] vs GMC, 19.4 [95% CI, 11.9–31.8]; *P* < .001). Figure 2 shows the GMCs for antibodies to TT, DT, PT, FHA, and Prn after the fourth aP-or wP-containing vaccine in the Tdap group.

## DISCUSSION

The present study describes the effect of maternal vaccination with a Tdap vaccine (Adacel) on the antibody titers in infants after a booster vaccination with an aP-containing vaccine during the second year of life. Previously, blunting of the infant immune response by maternal vaccination during pregnancy, in comparison with a control group receiving a tetanus-only vaccine during pregnancy, has been described for anti-DT and anti-Prn antibodies after a primary series of 3 aP-containing infant vaccines [4].

The present data indicate that a blunting effect by maternal immunization only persists on the anti-TT IgG titers in the Tdap group, 1 month after a fourth vaccine dose is offered in the second year of life, compared to the TT group. For anti-PT IgG, anti-FHA IgG, anti-Prn IgG, and anti-DT IgG, comparable but lower titers are measured in the Tdap group compared with the TT group. Nevertheless, a good humoral immune response is reported in both study groups, with a significant rise of antibody titers for all measured antigens, except DT-oriented antibodies, upon the fourth vaccine dose. The interval between vaccine dose 3 and 4 was significantly smaller in the TT group (16.58 months [SEM, 0.26 months] vs 17.44 months in the Tdap group [SEM, 0.17 months]; *P* = .01). But this was only affecting the anti-FHA antibody titers in the regression analysis.

In comparison with the available literature on general infant humoral immune responses to a booster dose of Infanrix Hexa in the second year of life [18, 19], some slight differences were found. Tichmann et al [18] collected blood samples after a fourth dose of Infanrix Hexa administered at 12–19 months of age. And Gimenez-Sanchez et al [19] collected blood samples after a fourth dose of Infanrix Hexa at 11–15 months of age, administered concomitantly with 7- or 13-valent pneumococcal conjugate vaccine. Different laboratory tests were used in both studies compared to this study. Antibody titers to anti-PT IgG, measured at 1 month after the fourth vaccine dose, are comparable [19] or higher [20] in the present study in both groups and antibody titers of anti-FHA IgG, anti-Prn IgG, and anti-DT IgG are lower in our study compared with both other publications [19, 20]. On the other hand, we report lower anti-TT antibody titers after the booster dose compared with the Tichmann et al study [19], but higher compared with the Gimenez-Sanchez et al study [20].

The clinical relevance of the lower antibody titers in children from vaccinated mothers after a fourth vaccine dose, yet rising titers compared to the post–primary time point within one study group, is a point of discussion, as no correlate of protection is known for pertussis. But high concentrations of anti-PT IgG and anti-Prn IgG are associated with protection against pertussis disease and mainly anti-PT antibodies are considered to be crucial for this protection [14, 20]. No clinical cases of pertussis were identified within our study population. However, in Vietnam, pertussis disease is only diagnosed based on a clinical definition. Laboratory diagnosis is not obtained because diagnostic equipment is not available at the community level. Therefore, underdiagnosis is highly probable. Antibody titers for tetanus and diphtheria remained above the correlate of protection both after primary and booster vaccination.

In the study performed by Muñoz et al [3], blunting of the antibody response after primary vaccination (at 2, 4, and 6 months) was also reported. This effect disappeared with the administration of a fourth vaccine dose at 12 months of age. Similarly, Hardy-Fairbanks et al [21] reported a slight blunting of the immune response after primary vaccination. Yet, after administration of a fourth vaccine dose at 12–18 months of age, no notable differences in antibody concentrations were encountered anymore between infant groups. In a Belgian study, a similar blunting effect on the immune response after primary vaccination (2, 3, and 4 months) was described [5]. After the administration of a fourth vaccine dose at 15 months of age, only a significant blunting effect remained for the anti-PT antibodies [11].

The differences observed between the present data and the studies described above [3, 5, 11, 21] could be due to the use of other vaccine brands in pregnant women or during infancy, to distinct primary vaccination schedules, to another timing of the administration of the fourth vaccine dose, different laboratory tests used [3, 21], or possible confounders between populations (eg, different demographic composition of the study population, different disease-specific epidemiological background, different vaccination history).

The blunting effect described is in contradiction with the observations in mice by Feunou et al where less blunting effect is described whenever different brands of vaccines are used in mothers and infants [22] compared with the same brand in mother and offspring. However, taking into consideration the small sample size of our study, the possible effect of the use of vaccines from several manufacturers certainly needs to be further investigated in future studies.

The linear regression model identified no persistent influencing factor on the antibody titers measured at 1 month after the fourth vaccine dose in our study population. Only single significant influences of some variables on 1 specific antigen at 1 specific time point were found (eg, sex on anti-DT IgG and interval between vaccine dose 3 and 4 on anti-FHA IgG).

The original design of this study was to vaccinate all participating infants with the wP vaccine used within the EPI. Due to previously described fatalities among young infants in Vietnam, and subsequent disruption of the national program, Infanrix Hexa was approved to be administered to all participating infants [4]. Then again, due to an unforeseen delay in the ethical approval of the booster dose administration within the study, 19 children overall (Tdap and TT group) received a fourth (booster) wP vaccine dose within the regular Vietnamese EPI services. This situation created the unique opportunity to report on different infant vaccination schedules after maternal immunization. Within the Tdap group, all measured antibody titers in wP-boosted infants were lower compared with the antibody titers in aP-boosted infants. For anti-PT IgG, these antibody titers were even significantly lower. These lower antibody titers could potentially be influenced by the longer interval between the fourth vaccine dose and blood sampling in the wP-boosted infants (see Supplementary Table 1 for details). Yet the difference in the anti-PT antibody titers is unlikely to be solely the consequence of the longer time lapse between booster vaccine and blood sampling. It is well known that higher antibody responses to aP vaccination are elicited compared with wP vaccination in infants after both primary and booster vaccination [23, 24].

### Limitations of the Study

Our study has a number of limitations. First, no blood samples were taken before the administration of the fourth vaccine dose. Consequently, we could not describe the antibody decay between the third and fourth vaccine dose.

Second, due to a delay in ethical approval, not all children were vaccinated with the same vaccine as a fourth vaccine dose. Some children were already vaccinated within the standard EPI healthcare system before ethical approval was obtained. However, these unforeseen circumstances offered the opportunity to investigate different schedules of boosting.

During the follow-up of the study, we experienced a dropout rate due to moving of participants to other provinces. The lower sample size of the study resulted in larger confidence intervals and lower statistical power, but we were still able to detect significant differences 1 month after the fourth vaccine dose.

## CONCLUSIONS

Pertussis vaccination during pregnancy closes adequately the susceptibility gap for infection in young unvaccinated infants. Previously, blunting of the infant immune response after 3 doses of an aP-containing vaccine has been reported for the anti-DT and anti-Prn antibody immune response in infants in Vietnam, when vaccination is performed in the presence of high titers of maternal antibodies after a 3-dose priming schedule. After the fourth dose with a pertussis-containing vaccine in the second year of life, significant blunting is reported for the anti-TT antibody immune responses. However, a strong humoral immune response on the fourth vaccine dose is elicited for all antigens, except DT, in both groups of infants from either Tdap- or TT-vaccinated women.

The data reported in this manuscript can add evidence for national and international decision makers on maternal immunization as a vaccination strategy for protection of young infants against infectious diseases. Further research on pertussis vaccination during pregnancy in low- and middle-income countries is certainly needed to assess the impact of high maternal antibody levels on the immune response of infants both primed with aP- or wP-containing vaccines. An additional comparative study on different brands of pertussis vaccines could shed further light on the induction of qualitative and quantitative differences between the induced immune responses.

## Supplementary Data

Supplementary materials are available at http://cid.oxfordjournals.org. Consisting of data provided by the author to benefit the reader, the posted materials are not copyedited and are the sole responsibility of the author, so questions or comments should be addressed to the author.

Supplementary Data
