# Supplementary material for: The Effect of Maternal Pertussis Immunization on Infant Vaccine Responses to a Booster Pertussis-Containing Vaccine in Vietnam
Source: Clin Infect Dis. 2016 Nov 2;63(Suppl 4):S197–204. doi: 10.1093/cid/ciw551 (PMC5106623; doi:10.1093/cid/ciw551)
Supplement: Supplementary Data [file supp_63_suppl-4_S197__index.html]

Supplementary Data 

# The Effect of Maternal Pertussis Immunization on Infant Vaccine Responses to a Booster Pertussis-Containing Vaccine in Vietnam

## Supplementary Data

Supplementary Data

- Supplementary Data - Pdf file
