# Supplementary material for: The Effect of Maternal Pertussis Immunization on Infant Vaccine Responses to a Booster Pertussis-Containing Vaccine in Vietnam
Source: Clin Infect Dis. 2016 Nov 2;63(Suppl 4):S197–204. doi: 10.1093/cid/ciw551 (PMC5106623; doi:10.1093/cid/ciw551)
Supplement: Supplementary Data [file supp_ciw551_ciw551supp.pdf]

Supplemental Table 1: Demographic characteristics of infants in the Tdap group receiving a fourth aP or wP containing vaccine dose

		Tdap group		p-value
		aP vaccinated infants	wP vaccinated infants	
**N (included infants)**		30	15	
Infant gender, No. (%)	Male	20 (66.7)	8 (53.3)	0.517
	Female	10 (33.3)	7 (46.7)	
Mean weight in kilograms (SEM)		10.75 (0.20)	10.77 (0.32)	0.963
Mean length in centimeters (SEM)		83.20 (0.59)	82.71 (0.87)	0.639
Mean age at vaccine dose 4 in months (SEM)		22.18 (0.27)	20.81 (0.43)	0.007
Mean age at blood sample 1 month after fourth vaccine dose in months (SEM)		23.18 (0.27)	23.98 (0.32)	0.065
Mean interval between vaccine dose 4 – blood sample 1 month after fourth vaccine dose in months (SEM)		0.99 (0.01)	3.16 (0.20)	<0.001
Mean interval between vaccine dose 3 and vaccine dose 4 in months (SEM)		17.44 (0.17)	15.24 (0.29)	<0.001
